# *REM34* and *REM35* Control Female and Male Gametophyte Development in *Arabidopsis thaliana*

**DOI:** 10.3389/fpls.2019.01351

**Published:** 2019-10-24

**Authors:** Francesca Caselli, Veronica Maria Beretta, Otho Mantegazza, Rosanna Petrella, Giulia Leo, Andrea Guazzotti, Humberto Herrera-Ubaldo, Stefan de Folter, Marta Adelina Mendes, Martin M. Kater, Veronica Gregis

**Affiliations:** ^1^Dipartimento di Bioscienze, Università degli Studi di Milano, Milan, Italy; ^2^Laboratorio Nacional de Genómica para la Biodiversidad, Unidad de Genómica Avanzada, Centro de Investigación y de Estudios Avanzados del Instituto Politécnico Nacional, Irapuato, Mexico

**Keywords:** gametophyte development, REM, transcriptional regulation, ovule, pollen, post-meiotic division, *Arabidopsis thaliana*

## Abstract

The REproductive Meristem (REM) gene family encodes for transcription factors belonging to the B3 DNA binding domain superfamily. In *Arabidopsis thaliana*, the *REM* gene family is composed of 45 members, preferentially expressed during flower, ovule, and seed developments. Only a few members of this family have been functionally characterized: *VERNALIZATION1* (*VRN1*) and, most recently, *TARGET OF FLC AND SVP1* (*TFS1*) regulate flowering time and *VERDANDI* (*VDD*), together with *VALKYRIE* (*VAL*) that control the death of the receptive synergid cell in the female gametophyte. We investigated the role of *REM34*, *REM35*, and *REM36*, three closely related and linked genes similarly expressed in both female and male gametophytes. Simultaneous silencing by RNA interference (RNAi) caused about 50% of the ovules to remain unfertilized. Careful evaluation of both ovule and pollen developments showed that this partial sterility of the transgenic RNAi lines was due to a postmeiotic block in both female and male gametophytes. Furthermore, protein interaction assays revealed that REM34 and REM35 interact, which suggests that they work together during the first stages of gametogenesis.

## Introduction

In higher plants, the alternation between the diploid sporophytic generation and the haploid gametophytic generation is a fundamental characteristic of their life cycle. The formation of the gametophyte from the sporophyte is the result of two sequential processes, sporogenesis, and gametogenesis. Angiosperms are heterosporous plants, characterized by the production of two types of unisexual gametophytes, the megagametophyte (embryo sac), and microgametophyte (pollen). Developments of both female and male gametophytes can be divided into two main steps: sporogenesis, during which meiosis occurs giving rise to haploid spores, and gametogenesis, which leads to the formation of the gametes (Berger and Twell, 2011).

In Arabidopsis, the female gametophyte develops in the gynoecium. The first step of megasporogenesis consists in the formation of the ovule primordia, in which one cell differentiates into the megaspore mother cell (MMC) or megasporocyte; the MMC sustains one meiotic division, giving rise to four haploid megaspores. Only one of them, the functional megaspore, continues its development and goes through three mitotic divisions forming a mature embryo sac composed of eight nuclei and seven cells: three antipodal cells, two medial polar nuclei, and one egg cell surrounded by two synergids (Mansfield and Briarty, 1991).

In the anthers, the microspore mother cell gives rise, through meiosis, to four microspores, which develop into mature pollen grains, containing two sperm cells surrounded by the vegetative cell (Hafidh et al., 2016).

The transition from sporogenesis to gametogenesis is directly correlated with the cell cycle transition from meiosis to mitosis. During gametogenesis, the number of mitotic divisions (two for the male and three for the female gametophyte) has to be tightly regulated and coordinated with cytokinesis. This cell division process is complex and requires the integration of different pathways such as those involved in cell cycle progression, chromatin modifications, and hormonal signaling. Moreover, mitotic progression during gametogenesis is also affected when interfering with basic biological processes like organelle and ribosome biogenesis (Shi et al., 2005; Li et al., 2009; Wang et al., 2012).

In both gametophytes, the retinoblastoma-related protein (RBR) plays a key role in the regulation of the cell cycle by inhibiting cell cycle entry through repressing E2F transcription factors. The *rbr* mutation results in an uncontrolled nuclear proliferation in both gametophytes (Ebel et al., 2004; Ingouff et al., 2006; Johnston et al., 2008). More recently, *RBR* was also associated with the meiosis activation, when the MMC is getting reduced by meiosis and forming subsequently the functional megaspore (Zhao et al., 2017).

In all eukaryotic organisms, cell cycle progression is tightly linked to the activation and degradation of different cyclin-dependent kinases (CDKs). During both female and male gametophyte developments, the activity of two homologous RING finger E3 ubiquitin ligases, RHF1 and RHF2, are required for the degradation of the CDK inhibitor ICK4/KRP6, which allows the correct progression of the cell cycle. In the *rhf1 rhf2* double mutant, both female and male gametophytes fail to complete their development and are arrested in FG1 and microspore stage respectively (Liu et al., 2008).

The transcriptional activity in different cell types during plant development is dependent on epigenetic modifications, such as chromatin remodeling and histone modifications. Failure in the establishment of such modifications can cause different defects throughout the plant’s life cycle. During gametogenesis, silencing of the *CHROMATIN-REMODELLING PROTEIN 11* (*CHR11*) within the embryo sac causes an arrest of nuclear proliferation from stage FG1 to FG5 (Huanca-Mamani et al., 2005). Furthermore, mutations in the histone acetyl transferase genes *HAM1* and *HAM2* cause an arrest in the early stages of both megagametogenesis and microgametogenesis (Latrasse et al., 2008).

Genetic studies have identified a large number of loci that control gametophyte development. Molecular cloning and characterization of some of them have revealed insights in sporocyte formation, meiosis/mitosis, and gametophyte development. Detailed phenotypic and molecular characterization of mutants remains a big challenge also because of the complication to work with such mutants, which often are partially sterile or even lethal (Muralla et al., 2011).

In the context of finding new players involved in the control of this process, the *REM* gene transcription factor family promises to be a good candidate since two of the four REMs that were functionally chatacterized, *VERDANDI* (*VDD or REM20*) and *VALKYRIE* (*VAL or REM11*), have a function in gametophyte development (Matias-Hernandez et al., 2010; Mendes et al., 2016). The other two members, *VERNALIZATION1* (*VRN1* or *REM5*) and *TARGET OF FLC AND SVP1* (*TFS1* or *REM17*), were shown to be involved in the control of flowering time (Levy, 2002; Sung and Amasino, 2004; Richter et al., 2019).

The expression patterns of *REM* genes were analyzed by Mantegazza et al. (2014) showing that the majority of the members of this family are preferentially expressed during flower and seed developments. Through this analysis, we identified *REM34*, *REM35*, and *REM36*, which are mainly expressed in the reproductive meristems but also throughout different stages of flower development. *REM34*, *REM35*, and *REM36* are located in a cluster, containing in total nine *REM* genes on the fourth chromosome of *Arabidopsis*. *REM34*, *REM35*, and *REM36* are very similar, which might indicate a possible functional redundancy.

Insertional mutants already analyzed for *REM34* and *REM36* are not complete knock-outs and showed no visible phenotype whereas no insertional mutants are available for *REM35* (Mantegazza et al., 2014). Since these genes are located in linkage on the *Arabidopsis* genome, it is also practically impossible to obtain multiple mutant combinations by crossing the available mutant lines.

Therefore, in this study, we investigated the role of *REM34*, *REM35*, and *REM36* through their simultaneous downregulation by RNA interference. Plants in which at least *REM34* and *REM35* were down-regulated showed an early arrest in the development of both female and male gametophytes. The process of mega/micro sporogenesis was not affected, and meiosis was taking place. However, subsequent mitosis was not occurring after spore formation, suggesting that these genes play a role in gametogenesis progression.

## Materials and Methods

### Plant Material and Growth Conditions

All experiments were performed in *Arabidopsis thaliana* ecotype Columbia-0 (Col-0). Plants were grown in a controlled environment at 20–22°C either under long day conditions (16 h light/8 h dark) or under short day (8 h light/16 h dark) conditions for 4 weeks after germination and then transferred to long day conditions. The *suf4-1 pSUF4*:*SUF4*-*GUS* seeds were donated by S.D. Michaels. Tobacco plants were germinated and grown at 20–22°C under long day conditions.

### RNA Interference and *35S:EAR_REM34* Constructs

To obtain the *REM_RNAi* construct 252, 232 and 254 base pairs long DNA fragments specific for the coding sequence of each of the genes *REM34, REM35*, and *REM36* were selected (the primers used to amplify the fragments are listed in the **Supplementary Table 1**). The fragments specificity was checked by BLAST against the Arabidopsis genome.

The three selected regions were PCR amplified, adding the *BsaI* sites to the primers, and cloned in a pENTR™ vector previously modified to function as a Golden Gate acceptor, with a single Golden Gate reaction, producing the pENTR-*RNAi_REM* vector. The Gateway LR reaction (Invitrogen™ Gateway™ recombination cloning system) was then performed to sub-clone the *RNAi_REMs* fragments into the pFGC5941 vector and used to transform Arabidopsis. Primers that were used are listed in **Supplementary Table 1**.

The EAR motif was added to the C terminus of the *REM34* coding sequence (see primer sequences in **Supplementary Table 1**). The fragment was cloned into the pB2GW7 plasmid (35S) passing through the pENTRY-D-TOPO vector (Invitrogen™ Gateway™ recombination cloning system). Arabidopsis plants were transformed using the floral-dip method (Clough and Bent, 1998).

### Quantitative RT-PCR

Total RNA was extracted from whole inflorescences. RNA samples were treated with DNase (TURBO DNA-free^®^; Ambion, http://www.ambion.com/) and retrotranscribed employing the ImProm-IITM Reverse Transcription System (Promega). Diluted aliquots of the cDNAs or genomic DNA were used as templates in qRT-PCRs, using the iQ SYBR Green Supermix (Bio-Rad) to detect target synthesis. All the experiments were performed with three technical replicates for each of the three biological replicates, with the exception of the expression analysis of *REM34, REM35*, and *REM36* in the T1 *REM_RNAi*, in the T1 35S:*REM34_EAR* plants and for T-DNA abundancy evaluation. Primers employed for these analyses are listed in **Supplementary Table 1**.

### Silique Length, Seed Number Evaluation, and Reciprocal Crosses

For each line, 10 siliques (dissected from three different plants) were measured, and seed, aborted seed, and non-fertilized ovule numbers were counted. For this purpose, a Leica^®^ MZ 6 microscope was used.

For the reciprocal crosses between wild-type and *REM_RNAi* #1 plants, mature siliques as well as open flowers and buds in an advanced stage of development were removed from the inflorescence of the mother plant, along with the meristem and smallest buds. Remaining buds were emasculated by removal of all floral organs except for the ovary. Then, anthers in the correct stage of development were taken from other flowers and used to pollinate the stigma. The numbers of seeds and unfertilized ovules were assessed for at least five pistils for each cross, and three biological replicas of the experiment were performed.

### *In Situ* Hybridization Analysis

*In situ* hybridization analysis for *REM34*, *REM35*, and *REM36* were performed following the same protocol and employing the same probes described by Mantegazza et al. (2014). Evaluation of the expression profile in the inflorescence and flower meristems was used as a positive control.

### Protein–Protein Interaction Analysis

Yeast two-hybrid assays were performed in the yeast strains PJ69-4A and PJ69-4α (de Folter and Immink, 2011). The coding sequences of *REM34*, *REM35*, and *REM36* were cloned in the pDEST32 (bait vector, BD; Invitrogen) and pDEST22 (prey vector, AD; Invitrogen) Gateway vector. The bait constructs were tested for autoactivation on selective yeast synthetic dropout medium lacking Leu, Trp, and His supplemented with 1, 3, 5, 10, or 15 mM of 3-aminotriazole, in order to set the screening conditions. After mating, colonies were plated on the proper selective media and grown for 5 days at 20°C.

The same coding sequences were also cloned in the pYFPN43 and pYFPC43 vectors, to perform the BiFC assay. Agrobacterium, transformed with the vectors and the viral suppressor p19 construct, was used to infiltrate tobacco leaves. The abaxial surfaces of infiltrated leaves were imaged 3 days after inoculation. As positive control for the infiltration, the already published VAL-VDD interaction was tested (Mendes et al., 2016). As negative controls, the constructs containing the proteins of interest were co-transformed with the empty pYFN43 and pYFC43 vectors. Furthermore, REM34 homodimerization, which was not observed in the Y2H assays, was also employed as a negative control (**Supplementary Figure 5**).

### Female Gametophyte Characterization

Female gametophytes were cleared and analyzed as previously described by Brambilla et al. (2007). Inflorescences were prepared for observation using the following protocol: flowers were emasculated and the next day harvested. The emasculated pistils were left O/N at 4°C in a 1:9 acetic acid:ethanol solution. Samples were rehydrated by subsequent washes with ethanol 90 and 70% and then incubated O/N at 4°C in clearing solution (160 g chloral hydrate, 50 g glycerol, and H_2_O to a final volume of 250 ml). Pistils at different developing states were separated from the other floral organs and opened to evaluate the female gametophyte morphology. For these experiments, a Zeiss Axiophot^®^ microscope equipped with differential interference contrast (DIC) optics was used.

### *In Vitro* Pollen Germination

For this experiment, the protocol published by Bou Daher et al. (2009) was followed applying minor modifications.

Pollen grains were plated on small glass plates, containing 2.5 ml of pollen germination medium [PGM:18% sucrose, 0.01% boric acid, 1 mM CaCl_2_, 1 mM Ca(NO_3_)_2_, 1 mM MgSO_4_, 0.5% agarose pH = 7]. The plates were incubated overnight at 22°C, with wet paper to maintain humidity. The next day, pollen germination and growth were evaluated with a Zeiss Axiophot^®^ microscope.

### Aniline Blue Staining

Flowers were emasculated and, after 24 h, pollinated. The pollinated ovaries were collected at two different time points: 5 and 24 h after pollination. Samples were overnight fixed and stained in absolute ethanol/glacial acetic acid 9:1, as previously described by Mori et al. (2006). Subsequently, they were transferred into a 8M NaOH solution for 1 h at 50°C. Finally, the carpels were washed twice with ddH_2_O for 10 min. The staining was performed with a modified aniline blue solution (aniline blue 2%, glycerol 1 M ddH_2_O) (Takeuchi and Higashiyama, 2016). Samples were stored at 4°C for 3 h or overnight. The observation was done under UV light (350–400 nm) with a Zeiss Axiophot^®^ microscope.

### Pollen DAPI Staining

Pollen was stained according to Park et al. (1998). Mature pollen was obtained by placing 3–4 open flowers in a microcentrifuge tube containing 300 µl of 4′,6-diamidino-2-phenylindole (DAPI) staining solution (0.1 M sodium phosphate (pH 7), 1 mM EDTA, 0.1% Triton X-100, 0.4 µg/ml DAPI high grade, Sigma). After brief vortexing and centrifugation, the pollen pellet was transferred to a microscope slide and observed with a Zeiss Axiophot^®^ microscope. Pollen at earlier stages of maturation was also analyzed by dissecting single anthers. Anthers were disrupted on microscope slides and squashed in DAPI staining solution (1 µg/ml) under a coverslip.

### GUS Staining

β-Glucuronidase (GUS) assays were performed as described by Resentini et al. (2017). Pistils at different developmental stages were dissected and fixed in acetone 90% and incubated O/N at 37°C. After staining, they were cleared using the protocol described above.

### Alexander Staining for Pollen Grains

Staining of pollen grains was performed as described by Peterson et al. (2010). After fixation (performed with 6 alcohol:3 chloroform:1 acetic acid), the anthers were placed on a microscope slide with a few drops of staining solution (10 ml 95% alcohol, 1 ml malachite green (1% solution in 95% alcohol), 50 ml distilled water, 25 ml glycerol, 5 ml acid fuchsin (1% solution in water), 0.5 ml orange G (1% solution in water), 4 ml glacial acetic acid, and distilled water (4.5 ml) to a total of 100 ml). Samples were analyzed with a Zeiss Axiophot^®^ microscope.

### CLSM Analysis

For confocal imaging, the Laser Scanning Confocal Microscope Nikon A1 was used. Inflorescences were fixed as described by Braselton et al. (1996). Samples were then excited using a laser (532 nm), and emission was detected between 570 and 740 nm.

## Results

### RNAi Mediated Silencing of *REM34, REM35, and REM36*

Since *REM34, REM35*, and *REM36* are very similar and in linkage, an RNA interference approach was adopted to investigate their role during reproductive development in Arabidopsis.

**Figure 1:**
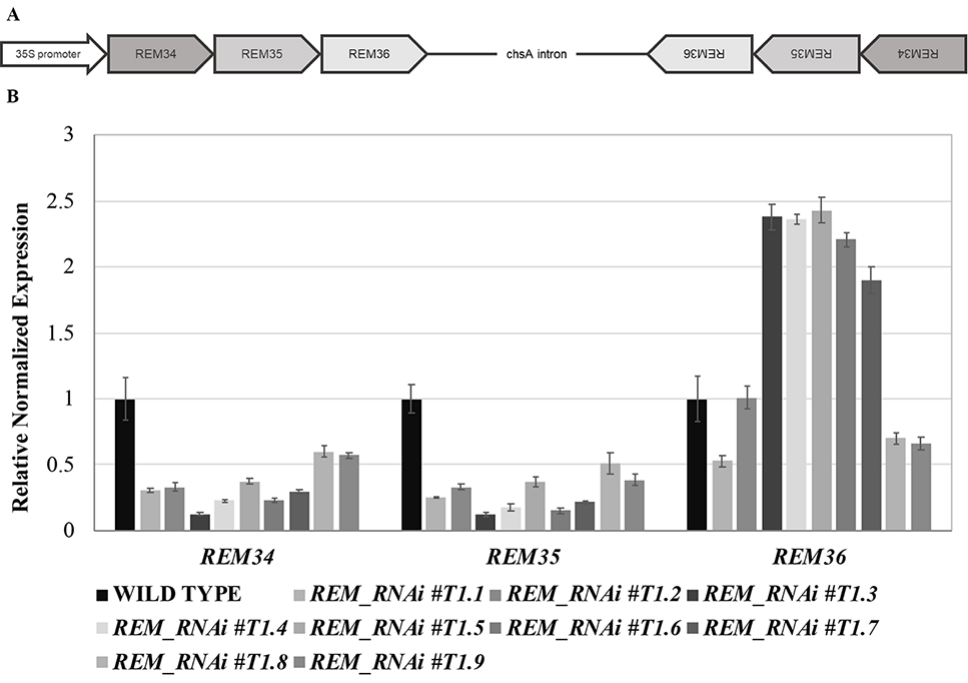
Multiple RNA interference lines. **(A)** Schematic representation of the RNAi construct. The REM34-REM35-REM36 sense and antisense fragments are separated by the chsA intron, to allow the hairpin structure formation. **(B)** qRT-PCR on nine different *REM_RNAi* T1 inflorescences, showing a strong downregulation of *REM34* and *REM35* and different levels of *REM36* expression.

Due to sequence divergency, even in the B3 DNA binding domain (Romanel et al., 2009), it was impossible to design a single artificial small interfering RNA fragment that was able to silence the three *REM* genes simultaneously. Therefore, a multiple RNA interference (RNAi) technology was used to express a single chimeric double stranded RNA that targeted the three REM genes under the control of CaMV35S (Miki et al., 2005; Bucher et al., 2006) (**Figure 1A**).

We selected three regions specific for the coding sequence of *REM34*, *REM35*, and *REM36*. The regions selected for *REM34* and *REM36* are highly specific for the genes of interest and were expected not to have any off target in the Arabidopsis genome. The RNAi fragment that targets *REM35* has a partial complementarity with *REM36*, and, at a lower level, with *REM37*, whose expression is almost undetectable in most Arabidopsis tissues (Mantegazza et al., 2014; Klepikova et al., 2016).

Forty *REM_RNAi* T1 transgenic *Arabidopsis* lines were obtained. We evaluated the down-regulation of the *REM* genes in nine different T1 lines (**Figure 1B**), which all showed defects in silique and gametophyte development.

Silencing of the three target genes was confirmed in the T2 generation by qRT-PCRs (**Supplementary Figure 1**). Furthermore, we showed that the RNAi construct was specific for their targets by testing the expression of *REM37* and *REM39*. The latter was chosen due to the fact that *REM39* is highly expressed in the tissues where *REM34, REM35*, and *REM36* are also active (Mantegazza et al., 2014; **Supplementary Figure 1**).

### *REM_RNAi* Lines Have a Reduced Ovule Number and Seed Set Compared to Wild-Type Plants

We selected three *REM_RNAi* lines (#1, #4, and #5), with different levels of silencing of *REM36*, for further investigations in the T2 generation. In line #1, *REM36* showed a downregulation of around 50%, while in lines #4 and #5, *REM36* was found to be slightly upregulated compared to the wild-type (**Figure 1B**).

**Figure 2:**
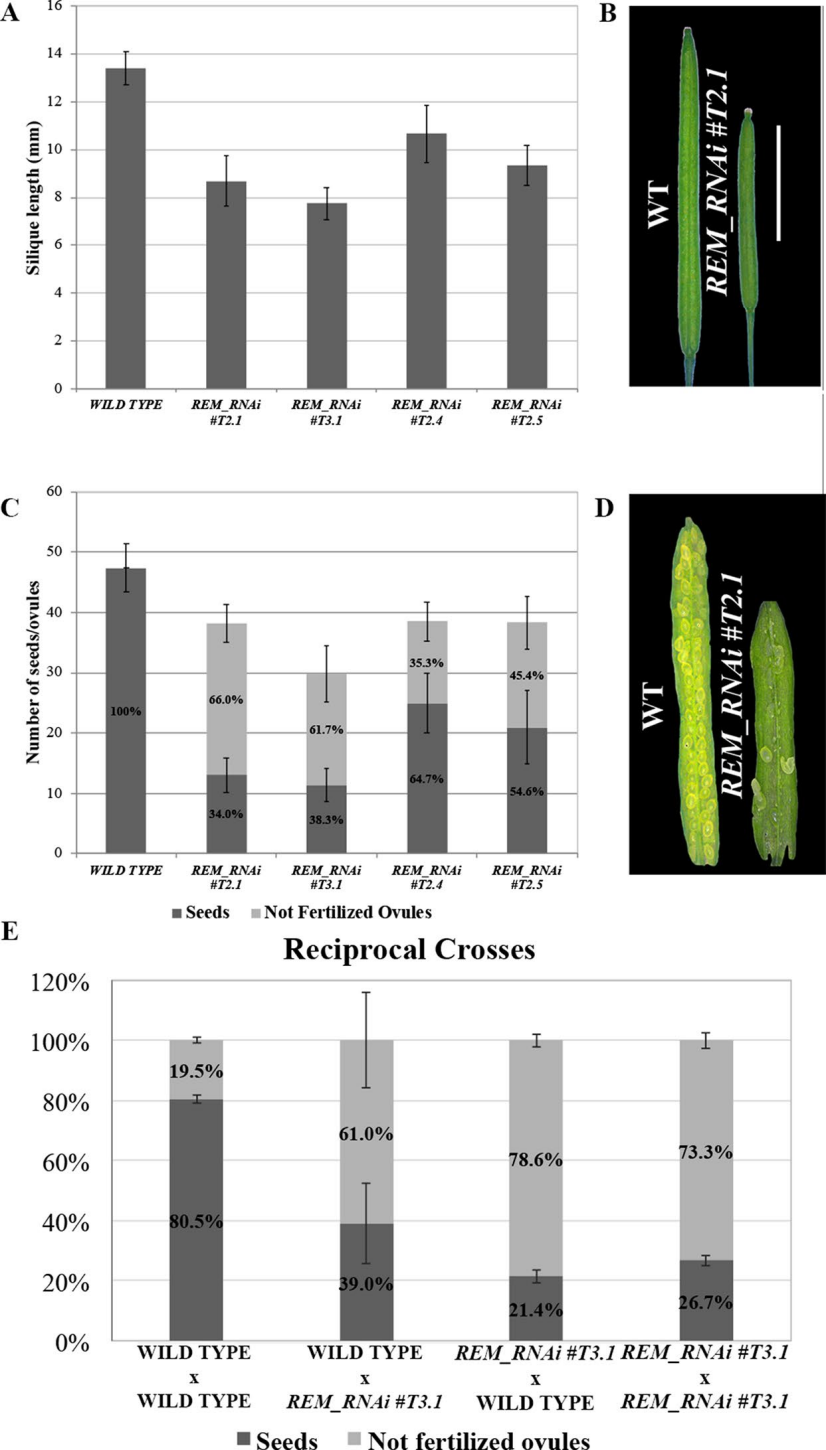
*REM_RNAi* lines have shorter siliques and a reduced seed set compared to the wild-type. **(A)** Graph showing the mean length of 10 wild-type and 10 *REM_RNAi #T2.1, #T3.1, #T2.4*, and #*T2.5* siliques. A wild-type silique measures on average 13.4 mm, the siliques from the different *REM_RNAi* lines were found to measure on average between 7.8 and 10.7 mm. (p < 0.01 for all comparison with the wild-type, ANOVA and *post hoc* Tukey HSD test were used). **(B)** Example of wild-type and *REM_RNAi #T2.1* siliques (bar = 5 mm). **(C)** Graph showing the mean number of ovules/silique in the wild-type and *REM_RNAi #T2.1, #T3.1, #T2.4*, and *#T2.5* plants, divided in seeds and not fertilized ovules. Compared to the wild-type situation, in which each silique contains on average 47.4 ovules, the *REM_RNAi* siliques have on average 29.8 to 38.5 ovules (p < 0.01 for all comparison with the wild-type, ANOVA and *post hoc* Tukey HSD test were used). On average between 35.3 and 66.0% of ovules, depending from the analyzed line, failed to be fertilized, while no aborted ovules were detected in the wild type situation (p < 0.01 for all comparison with the wild type, ANOVA and *post hoc* Tukey HSD test were used). **(D)** Example of wild-type and *REM_RNAi #T2.1* seed sets (bar = 5 mm). **(E)** Reciprocal crosses analysis between wild-type and *REM_RNAi #T3.1* plants. As a control, both wild-type x wild-type and *REM_RNAi #T3.1* x *REM_RNAi #T3.1* crosses were performed. Crosses are indicated female x male. (p < 0.01 for all comparison with the wild type of the non-fertilized ovules number, ANOVA and post hoc Tukey HSD test were used).

In the T2 generation, silique length and seed number were evaluated for the three selected lines. The *REM_RNAi #T2.1* line showed a decrease of 35.3% in the silique length and a 19.4% reduction in total ovule number (**Figures 2A–C**). Furthermore, on average 66% of the ovules failed to be fertilized (**Figures 2C, D**). The other two *REM_RNAi* lines, #*T2*.4 and #*T2*.5, showed a similar phenotype even if the percentage of unfertilized ovules was lower, 35.3 and 45.4%, respectively (**Figure 2C**).

The *REM_RNAi #1* line was selected to further investigate the sterility phenotype caused by the downregulation of *REM34, REM35*, and *REM36*. This line was propagated to the T3 generation, where plants homozygous for the *REM_RNAi* construct were selected. Even if the RNAi construct has a dominant effect, we evaluated whether the sterility observed in the *REM_RNAi T2* segregating lines was exacerbated in plants homozygous for the construct. For this purpose, the seed set of the *REM_RNAi T3.1* homozygous line was evaluated.

Interestingly, comparing both the *REM_RNAi #T2.1* and the *REM_RNAi #T3.1*, we noticed that the percentage of ovule abortion was the same, suggesting that the silencing of REMs is probably acting both at the sporophytic and gametophytic levels.

Since the two lines in which *REM36* was not downregulated displayed a milder phenotype compared to the *REM_RNAi #T2.1 and REM_RNAi #T3.1* lines, in which all three genes were downregulated, it is possible that *REM36* is partially redundant to *REM34* and *REM35* during gametophyte development. On the contrary, the ovule number was the same in all three *REM_RNAi* lines (**Figures 2A, C**), indicating that *REM36* is not involved in the determination of the ovule primordia number.

To further confirm that no phenotypical differences were detectable between plants homozygous and heterozygous for the T-DNA insertion, we analyzed the silique content of 10 *REM_RNAi #T2.4* and 10 *REM_RNAi* #*T2.5* T2 plants in which the construct was still segregating, and we found no significant differences between all the herbicide resistant plants (**Supplementary Figures 2A, C**). For both *REM_RNAi #T2.4* and *#T2.5* lines, a relative evaluation of T-DNA copies in each of the nine plants considered was performed. The RT-PCR analyses showed a various amount of T-DNA amplicons which is clearly unrelated to the ovule abortions and the overall seed set observed in all the *REM_RNAi #T2.4* and *#T2.5* analyzed individuals (**Supplementary Figure 2**). The *ACTIN7* amplicon was used as normalizer and the herbicide resistance BAR gene used to estimate the abundancy of T-DNA copies.

These analyses allowed excluding the possibility that the reduced seed set was linked to the presence of a heterozygous T-DNA insertion (Curtis et al., 2009; Clark and Krysan, 2010) and suggests that either the sporphytic silencing of *REM34*, *REM35*, and *REM36* affects the gametophyte or that the mobile siRNA diffuses from the sporophyte to the gametes (Mlotshwa et al., 2002; Melnyk et al., 2011; Skopelitis et al., 2018).

To understand if the reduced seed set was due to problems in the female or the male gametophyte, we performed reciprocal crosses between *REM_RNAi #T3.1* and wild-type plants. As a control, both *REM_RNAi #T3.1* (homozygous for the T-DNA triggering the RNAi silencing) and wild-type plants were manually selfed, in order to evaluate if the manipulation of the flower was affecting the fertility of the analyzed plants (**Figure 2E**).

When *REM_RNAi #T3.1* pistils were pollinated with *REM_RNAi #T3.1* pollen, 73.3% of the ovules failed to be fertilized while wild-type lines manually pollinated with wild-type pollen resulted in 19.5% unfertilized ovules. When the *REM_RNAi #T3.1* line pistils were pollinated with wild-type pollen, the percentage of unfertilized ovules was 78.6%, indicating a strong contribution of the female reproductive organ defects to this phenotype. Interestingly, when wild-type pistils were pollinated with *REM_RNAi #T3.1* pollen, still 61.0% of the ovules were not fertilized (**Figure 2E**). Moreover, we observed a high variability in the number of unfertilized ovules using *REM_RNAi* pollen as shown in **Figure 2E**. Macroscopical inspection revealed a decrease in pollen grain number compared to wild-type anthers and a lack of adherence of the pollen to the wild-type stigma, both observations were further investigated (see below). All these considerations strongly suggest that both female and male reproductive organs are affected in the *REM_RNAi* lines.

### *REM34, REM35*, and *REM36* Are Expressed in Both Female and Male Reproductive Organs in Adjacent Sporophytic and Gametophytic Cells

Previously, the expression pattern of the *REM* genes was characterized in the shoot apex by *in situ* hybridization analysis, showing that *REM34, REM35*, and *REM36* are expressed from the earliest stages of reproductive development of *Arabidopsis* in the inflorescence meristem and flower meristem and during the first stages of flower development with the exception of sepals (Franco-Zorrilla et al., 2002; Mantegazza et al., 2014).

In order to analyze the expression profiles in more detail during male and female sporophytic/gametophytic developments, we performed *in situ* hybridization analysis for *REM34, REM35*, and *REM36* in both female and male reproductive organs. The flower stages are described accordingly to Smyth et al. (1990) and Schneitz et al. (1995).

In Arabidopsis, pollen mother cells differentiate inside the young anther, ovule primordia arise from the placenta in stage 8 of flower development, and differentiation is completed at stage 13.

**Figure 3:**
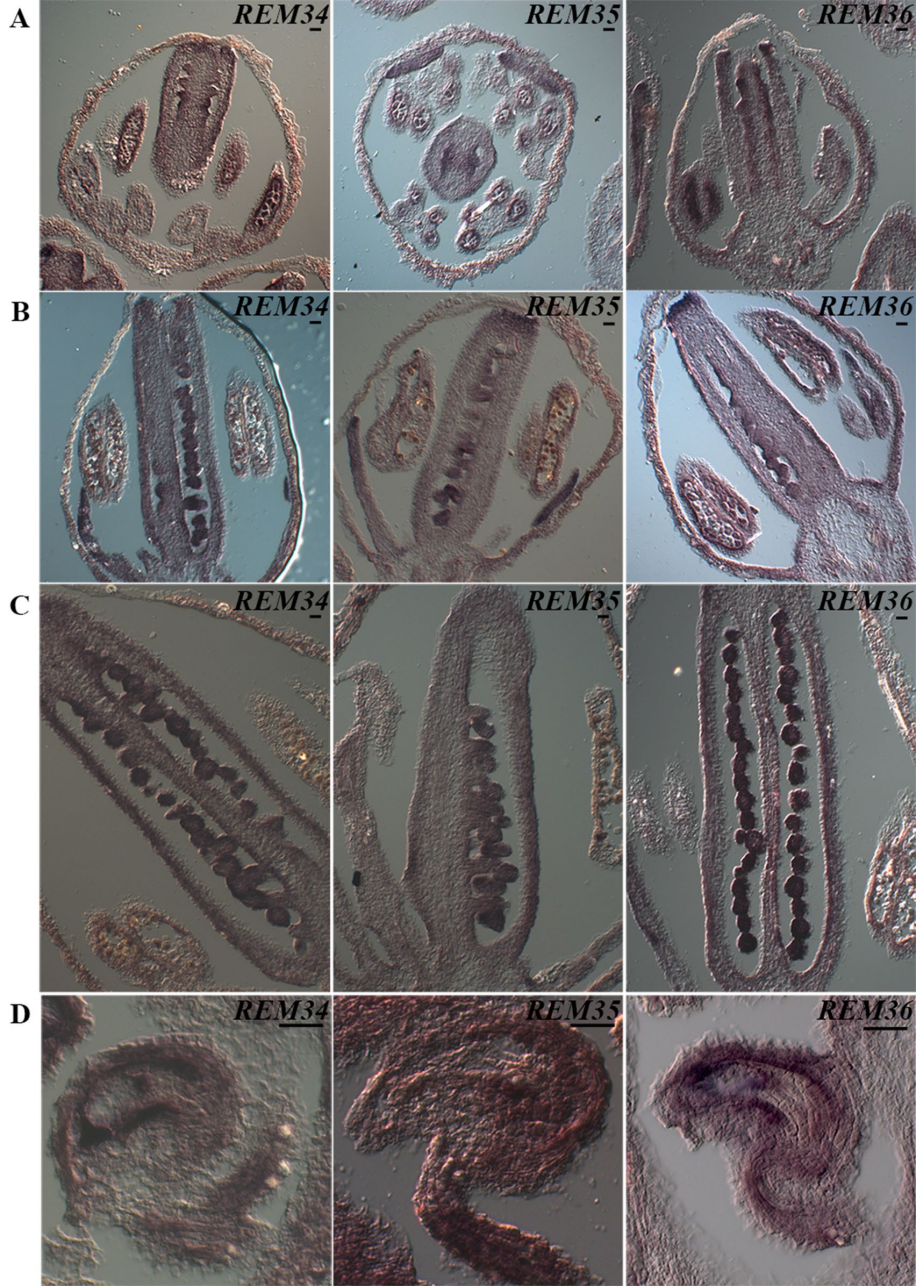
*REM34*, *REM35*, and *REM36* expression patterns. **(A)** In flowers at ST8-9, the signal in the carpel is restricted in the tissue of the placenta and ovules primordia. At the same stage, a clear signal is also visible in the anthers. **(B)** At ST10-11, the signal is present in the ovules, which are completing megagametogenesis, and in the anthers, where the pollen grains are undergoing the first mitotic division. **(C)** At ST12, when pollen reaches maturity, the signal is no longer visible in the anthers. **(D)** In flowers at anthesis, the target genes are expressed in the mature female gametophytes, in particular in the funiculus, inner integuments, and central cell. Flower stages are described accordingly to Smyth et al., 1990 (bar = 20 µm).

At stage 8/9 of flower development (Smyth et al., 1990), hybridization signals were detected for all three genes, in the anthers where the pollen mother cells differentiate and within the carpels, although in this case, the signal was stronger in the placenta and ovule primordia (**Figure 3A**).

At stage 10, a strong signal was always detected in developing ovules and pollen (**Figure 3B**). Our analysis revealed that the timing of expression of the three REM genes coincided with male and female sporogenesis.

During subsequent stages of flower development, stages 11–12, both female and male initiate gametophyte development. During these stages, a decrease in the signal was clearly observed in anthers (**Figure 3C**); during these stages, pollen reaches maturity, and the vegetative and generative cells are differentiated after mitosis (Park et al., 1998). In contrast, a strong signal was detected during ovule development when the surviving megaspore undergoes three rounds of mitosis and passes from stages 3I to 3V (Schneitz et al., 1995). Interestingly, when the ovule is at its very last stage of development 3-VI (Schneitz et al., 1995), a strong signal was detected in the funiculus, in the innermost integument and, inside the mature female gametophyte, in the central cell region (**Figure 3D**).

The expression analysis of *REM34, REM35*, and *REM36* highlighted the fact that, also during anther/pollen and carpel/ovule development, these three *REMs* have a similar pattern of expression.

The analysis of the expression patterns of *REM34, REM35*, and *REM36* combined with the phenotypes observed in the *REM_RNAi* lines denote an important role for these genes during the development and production of viable male and female structures and gametes.

### In *REM-RNAi* Lines the Female Gametophyte Is Unable to Complete Its Development

The expression profile of *REM34, REM35*, and *REM36* suggests that these genes play a role during ovule development. Furthermore, the reciprocal crosses showed that between 73.3 and 78.6% of the ovules in the *REM_RNAi #T3.1*, which is homozygous for the RNAi cassette, were not fertilized (**Figure 2E**).

**Figure 4:**
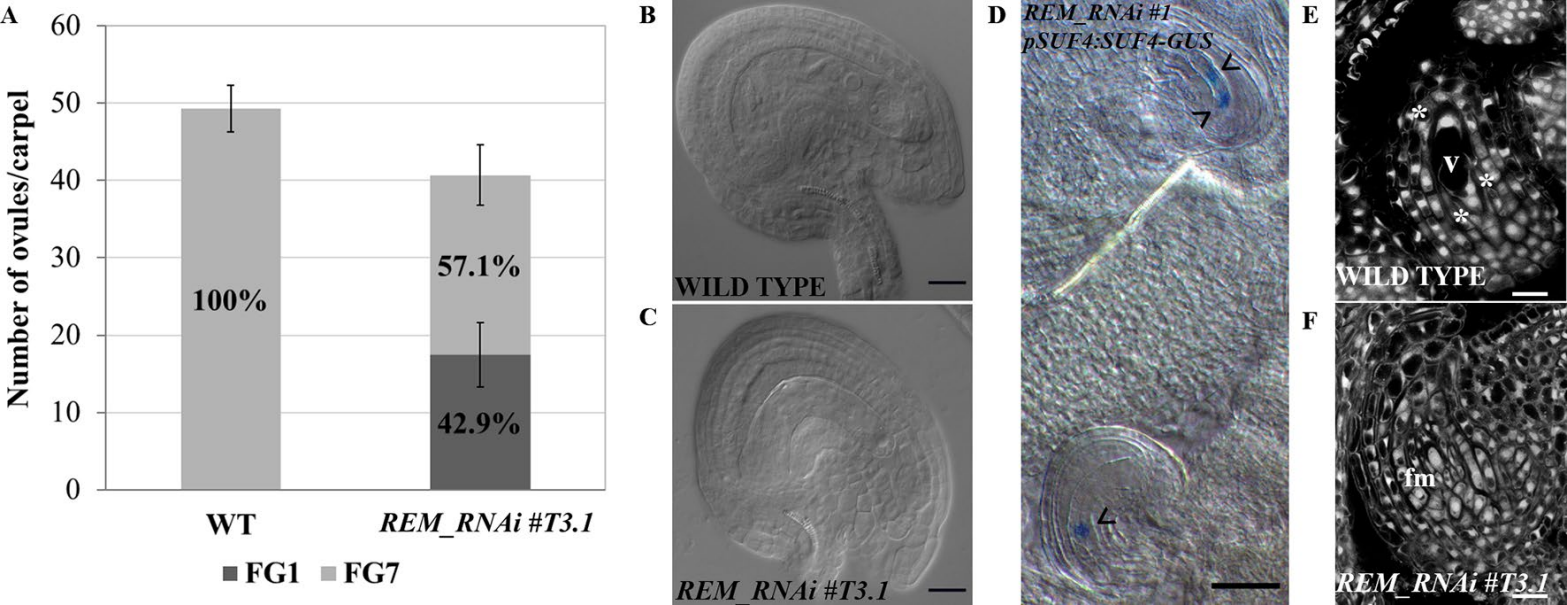
*REM_RNAi #T3.1* female gametophyte characterization. **(A)** Analysis of cleared mature carpels of both wild-type (n = 11) and *REM_RNAi #T3.1* (n = 13). In wild-type mature carpels, all the ovules reach the FG7 stage (542/542 ovules), while in the *REM_RNAi* line, 227/529 ovules are arrested at the FG1 stage. **(B)**, **(C)** Cleared ovules collected from both wild-type **(B)** and *REM_RNAi #T3.1* **(C)** mature carpels. In the wild-type situation, 100% of the embryo sac reaches the FG7 stage, while in the RNAi line, almost 60% of embryo sacs show an arrest in the FG1 stage (bar = 20 µm). **(D)** *pSUF4:SUF4-GUS* in the *REM_RNAi* line. In the uppermost ovule, two nuclei are stained, indicating the progression of gametogenesis till FG4 stage. In the lowest ovule 1, nucleus is stained indicating an arrest in FG1 stage. The arrowheads marked nuclei (bar = 50 µm). **(E**–**F)** CLSM analysis of *REM_RNAi #T3.1* ovules. In the same carpel, it was possible to observe ovules progressing in their development **(E)** and ovules arrested at the FG1 stage **(F)**; asterisks indicate three out of the four nuclei. v, vacuole; fm, functional megaspore (bar = 10 µm).

Based on this evidence, we hypothesized that the ovule defects in the *REM_RNAi* lines might be due to an arrest in their development. Therefore, a detailed evaluation of female gametophyte development was carried out in the *REM_RNAi #T3.1* homozygous line. In this line, 42.9% (227/529) of the ovules failed to complete their development and showed an arrest in the FG1 stage (**Figure 4A**). These ovules were characterized by an embryo sac containing one large cell, the functional megaspore, with a single nucleus; the rest of the ovules completed their development reaching the FG7 stage (**Figures 4B, C**). The same phenotype was observed in the *RNAi #T2.4* and *#T2.5* lines which both derived from hemizygous mothers (**Supplementary Figure 3**).

To confirm that, in the *REM_RNAi* lines, the defective female gametophytes were arrested in the FG1 stage, after meiosis, we crossed the *pSUF4:SUF4-GUS* marker line with the *REM_RNAi #T3.1* line. In the *pSUF4:SUF4-GUS* marker line, GUS expression is not detectable during megasporogenesis, but it becomes visible after meiosis, once the functional megaspore is formed, and marks all the nuclei of the embryo sac (Resentini et al., 2017). Observing *REM_RNAi #T3.1* pistils, both wild-type like ovules, with more than one nucleus and ovules arrested in the FG1 stage, with the nucleus of the functional megaspore, expressed the *GUS* reporter (**Figure 4D**) suggesting that the defect in female gametophyte development was post-meiotic.

To investigate in detail the arrest at the FG1 stage, we carried out confocal laser scanning microscopy (CLSM) on *REM_RNAi #T3.1* developing ovules. The feulgen staining perfectly marked the cell wall of the ovule integuments and the embryo sac dividing nuclei, allowing the recognition of the gametophytic developmental stages. In the same *REM_RNAi #T3.1* pistil, we observed ovules that normally developed until stage FG4 (**Figure 4E**) and those that were arrested in FG1 in which the embryo sac contains the functional megaspore and the three degenerating spores on top of it (**Figure 4F**).

### The *REM-RNAi* Lines Showed a Post-Meiotic Defect of the Male Gametophyte

**Figure 5:**
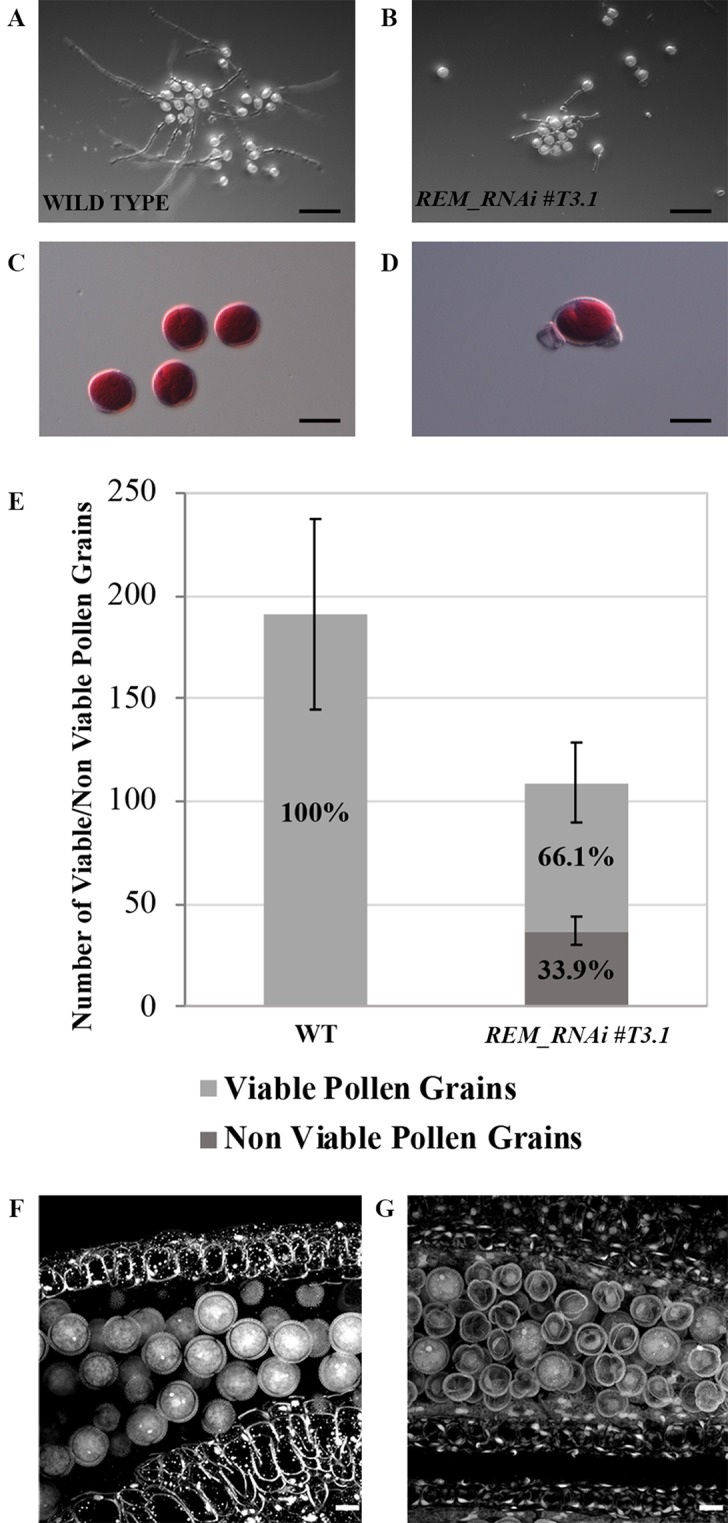
*REM_RNAi #T3.1* male gametophyte characterization. **(A**–**B)** *In vitro* germination test of wild-type **(A)** and *REM_RNAi #T3.1* **(B)** pollen grains, plates were imaged 24 h after plating (bar = 100 µm). **(C**–**D)** Pollen grains, collected from mature anthers of both wild-type **(C)** and *REM_RNAi #T3.*1 anthers **(D)**, were stained with Alexander’s staining to check pollen grain viability. While all the wild type grains were viable, some *REM_RNAi#T3*.1 pollen grains appeared shrunken and unable to be stained in red (bar = 20 µm). **(E)** Mature anthers from wild-type and *REM_RNAi#T3.1* flowers were dissected, the released pollen was collected and treated with Alexander’s staining to discriminate between viable and non-viable pollen grains. In the wild type, 100% of the pollen grains resulted vital while 33.9% of *REM_RNAi#T3*.1 pollen was found to be non-vital. (wt n = 1,337, *REM_RNAi#T3*.1 *= 874*; p < 0.01 for all comparison with the wild-type, ANOVA and *post hoc* Tukey HSD test were used). **(F**–**G)** CLSM analysis of wild-type **(F)** and *REM_RNAi#T3*.1 **(G)** mature anthers. All the wild-type grains are round and contain the vegetative nucleus and the two sperm cell nuclei; in the *REM_RNAi#T3.1* anthers, it is possible to visualize both pollen grains at two nuclei stage, as well as degenerate pollen grains, without any visible nucleus (bar = 10 μm).

From the analysis of wild-type carpels pollinated with *REM_RNAi* pollen, we observed that 61.1% of the ovules were not fertilized, suggesting that the male gametophyte in these lines is also defective (**Figure 2E**). To understand the cause of this defect, we first carried out an *in vitro* pollen germination assay which showed a 30% decrease in the germination rate of the *REM_RNAi #T3.1* pollen compared to the wild type (**Figures 5A, B** and **Supplementary Figure 4**).

The growth rate of *REM_RNAi* pollen tubes and their ability to correctly target ovules were also evaluated *in vivo* by means of aniline blue staining (**Supplementary Figure 4**). The *REM_RNAi* pollen tubes did not show any growth defect, they reached the end of the pistil in the same time as the wild-type pollen tubes, and the mature ovules were correctly targeted (**Supplementary Figure 4**). We noticed that, as mentioned before, the *REM_RNAi* pollen number appeared to be lower, and it did not adhere well to the stigma papillae, which could explain the high variability observed in the backcrosses between wild-type pistils and *REM_RNAi* pollen (**Figure 2E**).

To try to understand the cause of the male sterility phenotype, pollen grains were collected from mature anthers and treated with Alexander’s stain, which colors viable pollen red. While in the wild type, all the collected pollen was viable; in the *REM_RNAi #T3.1* anthers, 33.9% of the grains were not stained, indicating that those pollen grains were non-viable and did not appear to contain any cytoplasm (**Figures 5C–E**). Interestingly, the percentage of non-viable pollen grains in the *REM_RNAi* line corresponds to the decreased germination capability observed *in vitro*, suggesting that the grains which are unable to produce the pollen tube are the degenerated ones.

To investigate the pollen defect in more detail, confocal laser scanning microscopy (CLSM) was used. In **Figure 5F**, wild-type pollen from a mature anther is shown; intine and exine layers were very well distinguishable and inside the pollen grain, and the sperm cells and the vegetative cell nuclei were stained. On the contrary, in the *REM_RNAi #T3.1* mature anthers, a high percentage of pollen grains appeared shrunken and empty; neither sperm nor vegetative cells were identified, although the intine and exine layers looked intact (**Figure 5G**).

**Figure 6:**
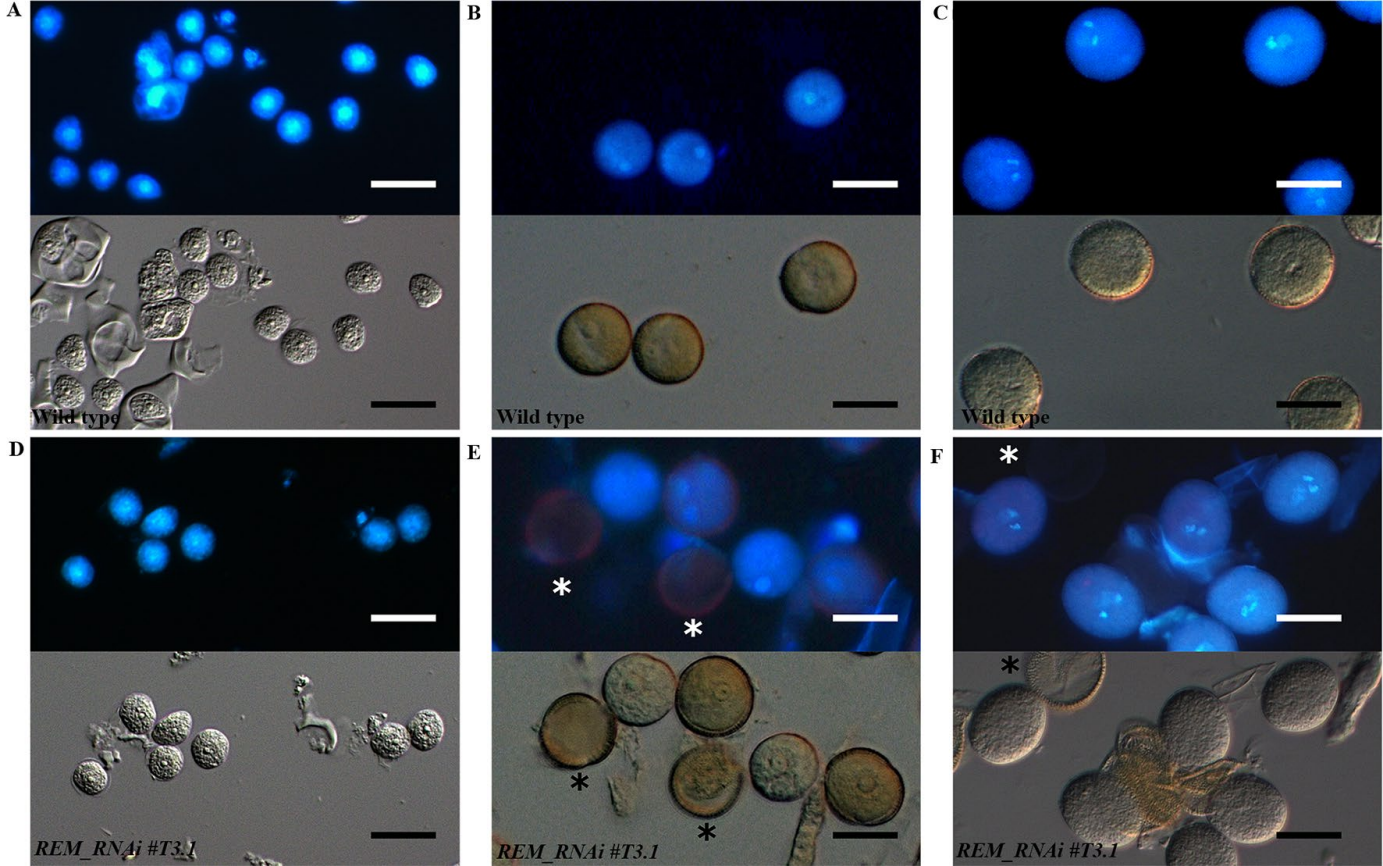
Wild type and *REM_RNAi* pollen development. DAPI staining of wild-type and *REM_RNAi#T3*.1 pollen grains at different developmental stages. At the microspore stage **(A** and **D)**, all the grains contain a well-defined central nucleus. At the bicellular stage in all wild-type grains **(B)**, the spermatic and vegetative nuclei are distinguishable, while in the *REM_RNAi#T3*.1 lines **(E)**, some grains, marked with an asterisk, do not display any nucleus. Wild-type mature pollen grains **(C)**, characterized by the presence of two sperm cells and one vegetative nucleus. *REM_RNAi#T3*.1 mature pollen **(F)**, the asterisk marks a mutant pollen grain without nucleus (bar = 10 µm).

To understand when the pollen grains degenerated, we visualized their nuclei with DAPI staining at different developmental stages (**Figures 6A–F** and **Supplementary Figure 3**). At the microspore stage, all *REM_RNAi #T3.1* grains were characterized by the presence of a single bright nucleus localized at the center of the cell, indicating that the pollen, like wild-type, passed through meiosis correctly (**Figures 6A, D**). After meiosis, in wild-type, the microspores underwent a first mitotic division that produced one vegetative and one sperm nuclei (**Figure 6B**). Subsequently, the second round of mitosis led to the formation of the mature pollen grain, which contained two small sperm cells each with a bright and elongated nucleus and the vegetative cell (**Figure 6C**). Interestingly, in *REM_RNAi #T3.1* anthers, some grains were characterized by the lack of nuclei; this phenotype was detectable also at the tricellular stage (**Figures 6E, F** and **Supplementary Figure 3**).

Thus, after meiosis, *REM_RNAi* anthers displayed both viable pollen grains, with two sperm cell nuclei and a distinct vegetative nucleus, and not viable pollen grains, in which no DNA is detectable (**Figures 6E, F**). This is similar to what was observed with the CLSM analysis.

All this evidence suggests that the degeneration of pollen grains observed in the *REM_RNAi* lines could be due to a post-meiotic block in their development, a similar defect as the one observed in the female gametophytes.

### REM35 Formed Homodimers and Heterodimers With REM34

**Figure 7:**
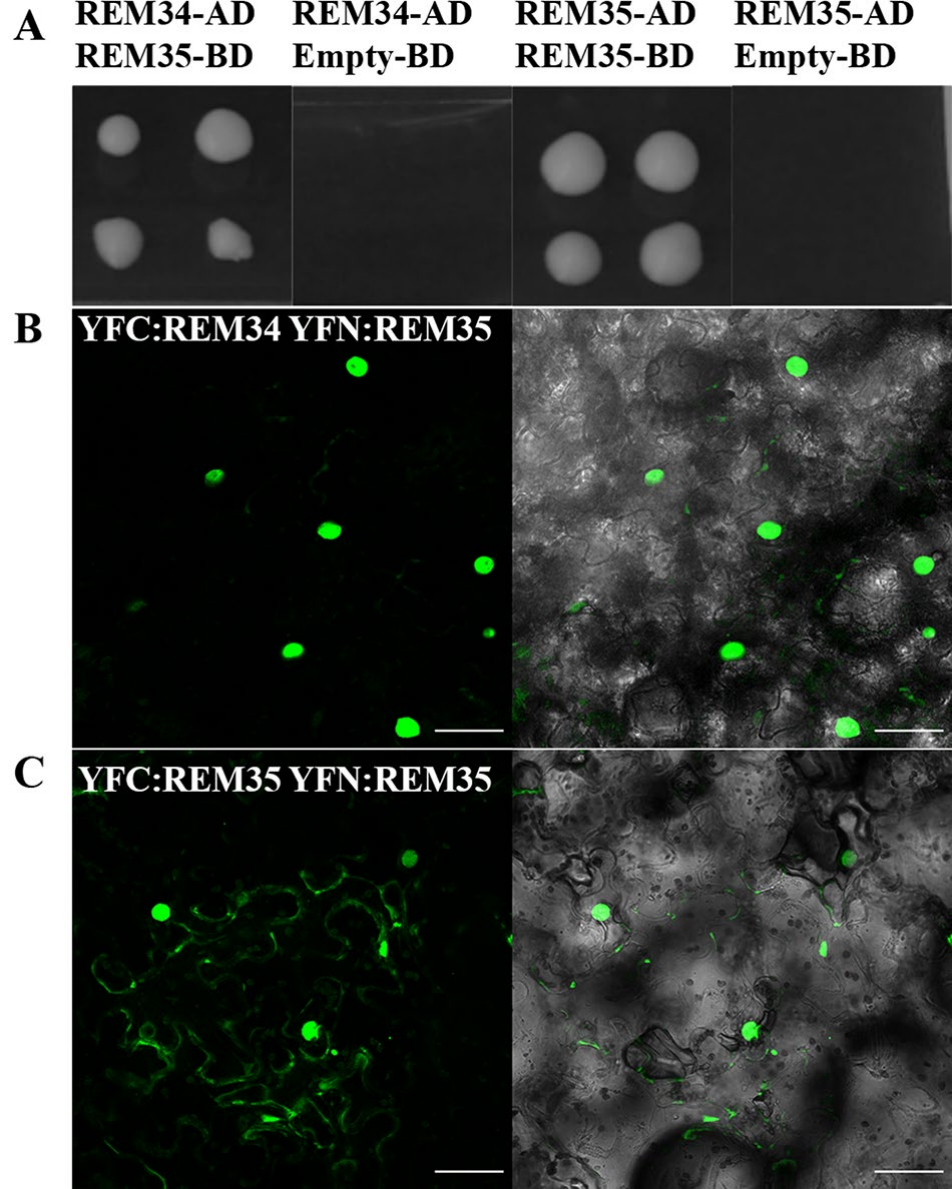
REM34 and REM35 interaction. **(A)** Yeast two hybrid assays showing the interactions between REM34 and REM35 and REM35 and REM35, on –L-W-H + 2.5 3-AT selective media. Empty pDEST32 vector was employed as a negative control. **(B**–**C)** BiFC experiments in tobacco leaf cells showing the reconstitute YFP fluorescence (green) between **(B)** REM34 and REM35 fusions to the C- and N-terminal fragments of YFP, respectively. **(C)** REM35 fusions to the C- and N-terminal fragments of YFP (bar = 50 µm).

REM transcription factors can form functional heterodimers (Mendes et al., 2016). To understand if also REM34, REM35, and REM36 could function *via* dimer formation, yeast two-hybrid assays were performed. This approach revealed that REM35 is able to interact strongly with itself and also with REM34, while no interactions were detected with REM36 (**Figure 7A** and **Supplementary Figure 5**).

All the interactions observed in the yeast two-hybrid assays were confirmed *in vivo* with a Bimolecular Fluorescence Complementation (BiFC) assay in *Nicotiana benthamiana* leaves (**Figures 7B, C** and **Supplementary Figure 5**). This finding suggests that REM34 and REM35 could act as heterodimers.

### Downregulation of *REM34, REM35*, and *REM36* Altered Expression of Genes Involved in Post-Meiotic Divisions

As described above, the downregulation of *REM34*, *REM35*, and partially *REM36* resulted in a post-meiotic arrest in both female and male gametophytes, suggesting that these transcription factors could be involved in regulating mitosis progression during gametogenesis.

To elucidate the molecular mechanism causing this block, we measured the expression levels of genes that control gametogenesis by q-RTPCR. We focused on genes that, when mutated or overexpressed, cause similar defects to those observed in the *REM_RNAi* gametophytes. Those genes were divided into three categories based on the biological process in which they are involved in: ribosome biogenesis (*MDS, NLE*), cell cycle control (*RBR, KRP6*), and chromatin regulation (*HAM1, HAM2*).

*MDS*, which, together with *NLE*, is involved in the biogenesis of the 60S ribosomal subunit and is essential during megagametogenesis (Chantha et al., 2010), was downregulated in the *REM_RNAi #T3.1* lines. *KRP6*, a CDK inhibitor whose overexpression causes a block in mitosis progression during female and male gametophytic development, was also downregulated in the *REM_RNAi* lines.

**Figure 8:**
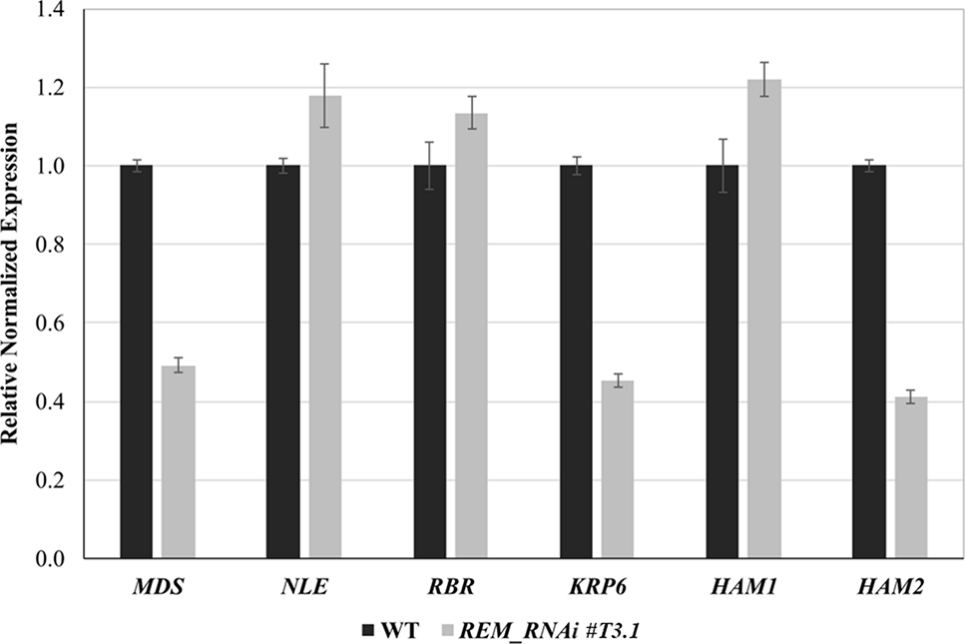
Expression analysis of genes involved in gametophyte development by qRT-PCR. Selected gene expression in inflorescence of wild-type and *REM_RNAi #T3.1*. The expression of selected genes was normalized to that of UBI, and the expression level in Col was set to 1.

Among the genes involved in chromatin modifications, two histone acetyltransferases (HATs), *HAM1* and *HAM2*, were selected. Only *HAM2* was downregulated in the *REM_RNAi #T3.1* line (**Figure 8**).

These results suggest an intricate interconnection among regulators and effectors, which end up in a correct gametogenesis program.

### Overexpression of the REM34_EAR Chimeric Protein

**Figure 9:**
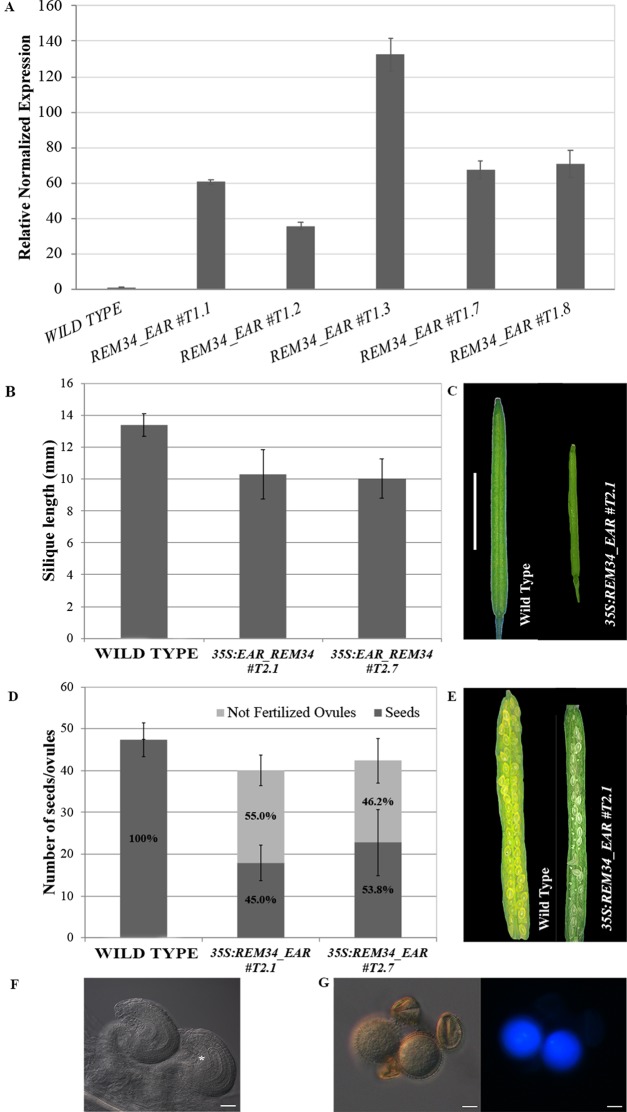
Analysis of the *35S:REM34_EAR* lines. **(A)** qRT-PCR for the evaluation of the *REM34_EAR* overexpression in five T1 transgenic lines. **(B)** Graph showing the mean length of 10 *REM34_EAR #T2.1 #T2.7* siliques, compared to the wild-type, the two lines have a reduction in the silique length (p < 0.01 for all comparison with the wild type, ANOVA and *post hoc* Tukey HSD test were used). **(C)** Example of wild-type and *REM34_EAR #T2.1* siliques (bar = 5 mm). **(D)** Graph showing the mean number of ovules/silique in the wild-type and *REM34_EAR #T2.1 #T2.7* plants. Both lines were characterized by a reduction in the total seed set of around 10% compared to the wild-type (p < 0.01 for all comparison with the wild type, ANOVA and *post hoc* Tukey HSD test were used). On average between 55.0 and 46.2% of ovules, depending from the analyzed line, failed to be fertilized, while no aborted ovules were detected in the wild type situation (p < 0.01 for all comparison with the wild type, ANOVA and *post hoc* Tukey HSD test were used). **(E)** Example of wild-type and *REM34_EAR #T2.1* seed sets (bar = 5 mm). **(F)** Cleared ovules sampled from mature *REM34_EAR #T2.1* carpels, the asterisk marks the one blocked at the FG1 stage (bar = 20 µm). **(G)** DAPI stained pollen grains, sampled from mature *REM34_EAR #T2.1* anthers; some grains were able to reach the tricellular stage and showed fluorescent nuclei while others appeared degenerated and with no visible nucleus (bar = 20 µm).

The genes that were downregulated in the *REM_RNAi* lines might be targets of the REM transcription factors. This suggests that REM34 and REM35 might be transcriptional activators. To investigate whether REM transcription factors work as activators of transcription, we fused REM34 with the dominant EAR repressor domain (known as chimeric repressor silencing technology CRES-T) and transformed wild-type *Arabidopsis* plants with this construct. Five transgenic lines that overexpressed the *REM34_EAR* chimeric gene at different levels in the T1 generation were obtained (**Figure 9A**).

In the T2 generation, silique length and ovule number were measured in two independent lines (*REM34_EAR*#T2.1 and *REM34_EAR*#T2.7). In both the selected T2 *REM34_EAR* lines, we observed a decrease in the silique length of 23.1 and 25.0%, respectively, and the presence of 55.0 and 42.6% aborted ovules, similar to what was observed in the *REM_RNAi* lines (**Figures 9B–E**).

The phenotype of the aborted ovules was further evaluated in cleared mature carpels of the *REM34_EAR #T2.1* and *#T2.7* lines. We detected both ovules at FG7 stage, with the seven cells clearly distinguishable, and ovules at FG1 stage, characterized by a single cell embryo sac (**Figure 9F**).

To confirm also the post meiotic block in the male gametophyte, mature pollen of both *REM34_EAR #T2.1* and #*T2*.*7* lines were stained with DAPI. Similarly to what was observed in the *REM_RNAi* lines, some pollen grains were able to reach the tricellular stage while others appeared shrunken and degenerated, with no visible nuclei (**Figure 9G**).

The strong similarity between the *REM34_EAR* and the *REM_RNAi* phenotypes might suggest that the overexpression of the chimeric REM34_EAR protein was causing co-suppression of other *REM* genes. To exclude this possibility, we investigated the expression level of *REM35, REM36, REM37*, and *REM39* in the *REM34_EAR #T2.1* and #*T2.7* lines. The level of expression of the endogenous *REM34* was not taken into account, as the perturbation of *REM34* expression alone did not cause any evident phenotypical defects (**Supplementary Figure 6**) (Franco-Zorrilla et al., 2002; Mantegazza et al., 2014). The obtained results showed that the closely related *REMs* were not affected suggesting that the expression of the REM34_EAR chimeric protein caused the observed phenotypes.

## Discussion

### Functional Analysis of *REM* Genes

The plant-specific REM family in Arabidopsis is composed of 45 genes, generated through multiple duplication events, which are mostly expressed during flower and ovule development (Romanel et al., 2009). Even if the expression pattern of these genes suggests that they could play an important role in regulating developmental processes such as shoot architecture and flower development, until now, only a few of them have been associated to a function (Levy, 2002; Matias-Hernandez et al., 2010; Mendes et al., 2016). This might be due to their functional redundancy but also because they are often in linkage on the genome.

Here, we investigated the function of the linked duplicated *REM34, REM35*, and *REM36* genes by a multiple RNAi approach and showed that *REM34*, *REM35*, and partially *REM36*, are involved in male and female gametophytic developments during post-meiotic divisions. A similar multiple RNAi approach was previously employed to silence simultaneously up to six target genes in *Arabidopsis thaliana* (Czarnecki et al., 2016).

The *REM_RNAi* construct was found to be a very efficient tool: by selecting specific gene sequences, we were able to silence the three target genes with a single construct and a single transformation event. Importantly, the construct showed to be highly specific for the three genes of interest without any obvious off-target activity. Transgenic lines showing silencing of the *REM* genes under study were all characterized by a reduced seed set and an arrest in female gametophyte development at the earliest stages of gametogenesis. Since *REM34, REM35*, and *REM36* appeared to be mainly expressed in sporophytic tissues throughout Arabidopsis reproductive development, the CaMV35S promoter was chosen to drive the expression of the RNAi fragments. The activity of the CaMV35S promoter seems to be low during female and male gametophyte developments, but it has been shown that such promoter can be successfully employed to silence genes during gametophytic development (Acosta-García and Vielle-Calzada, 2004; Mendes et al., 2016). A valid hypothesis for the observed gametophyte phenotypes might be that it is caused indirectly by the silencing of *REM34, REM35*, and *REM36* in the female and male sporophytic cells. However, it is also important to consider that the RNAi construct is dominant and that it can trigger a non-cell autonomous and systemic silencing signal which might be maintained throughout the different phases of plant development (Mlotshwa et al., 2002; Melnyk et al., 2011; Skopelitis et al., 2018).

Since functional redundancy is a common phenomenon in plants (Briggs et al., 2006), this kind of RNAi approach will be helpful for the functional characterization of members of highly redundant families and especially that are in linkage. Furthermore, since silencing of genes by *RNAi* is often not complete, this approach could favor the analysis of genes for whom knock-out leads to lethality or complete sterility.

### REM Protein Interactions

Protein interaction studies revealed that REM34 and REM35 were able to interact with each other; while no interaction was found with REM36, this supports the hypothesis that *REM36* might not be able to substitute completely *REM34* and *REM35* functions. Interactions between REM factors were found before. VDD and VAL, two functionally characterized REM factors involved in synergid degeneration upon fertilization (Mendes et al., 2016), also interact with each other. Furthermore, both *VAL* and *REM35* were also able to make homodimers. These characteristics might well be a common feature for the REM family and, in perspective of the guilt-by-association principle, it would be informative to analyze all possible REM protein interactions. The same approach was shown to be extremely useful for the characterization of MADS domain transcription factor family, for which extensive protein–protein interaction studies effectively guided genetic studies and functional characterization of many of them (de Folter et al., 2005; Gregis et al., 2006; Fornara et al., 2008; Immink et al., 2009).

### *REM34* and *REM35* Control Female and Male Gametogenesis

We discovered that, in the *REM_RNAi* lines, both the male and female germ lines were able to go through meiosis correctly, but they were not able to pass the FG1 stage, suggesting a role for *REM34* and *REM35* in the control of gametogenesis in Arabidopsis.

Although the *REM* gene family was named after the specific meristematic expression of its first member *AtREM1*, which was named *REM34* (Franco-Zorrilla et al., 2002), our data showed that *REM34*, *REM35*, and *REM36* are also expressed during gametophytic development, and we discovered that they were expressed starting from both carpels and anther primordia specification throughout all the stages of anther and carpel developments. In the carpel, the signal is strongly localized in the placenta and ovule primordia and in the developing ovules.

Indeed, our deep morphological analysis of both female and male gametophytes of the *REM_RNAi* lines showed that from 35 to 65% of the female gametophytes were unable to undergo mitosis and were arrested at the FG1 stage when the MMC acquires functional megaspore identity.

*REM36* seemed to be partially redundant with *REM34* and *REM35*. Indeed, in the two lines in which the level of *REM36* expression was higher compared to the wild-type, the penetrance of the embryo sac defect was less. However, in all *REM_RNAi* lines, we also observed a decrease of around 20% in the total ovules number irrespectively of the expression levels of *REM36*. Thus, *REM36* might be involved in embryo sac development together with *REM34* and *REM35* but is not controlling ovule primordia specification.

In these lines, also pollen development was affected showing the same post meiotic arrest of the embryo sac. Thus, in Arabidopsis, REM34, REM35, and partially REM36 transcription factors seem to be required post-meiotically for gametophytic development.

Further confirmation for their role during both female and male gametogenesis came from the analysis of different *35S:REM34_EAR* lines. These plants, in which *REM34* fused to the EAR repressor domain was overexpressed, showed the same postmeiotic arrest both in embryo sac and pollen development, suggesting that a complex formed by REM34 and REM35 could act as a positive transcriptional regulator of gametogenesis.

Because of the redundancy and position in linkage of the three genes of interest in the genome, most of this functional study was conducted using RNAi. This approach was found to be very effective in the silencing of *REM34, REM35*, and *REM36*, but the transgenic lines cannot be easily employed for genetic studies, due to the fact that it acts dominantly and because the level of silencing of the target genes can vary between different lines and throughout subsequent generations. Despite these difficulties, the analyses performed on both segregating and homozygous lines suggest that these three genes can influence gametogenesis acting mainly at the sporophytic level. This hypothesis is also supported by the expression pattern of these genes which, as shown by the *in situ* hybridization analysis, are present in the sporophytic tissues both in pistils and anthers when gametogenesis is taking place. The observation that *REM34, REM35*, and *REM36* appeared to be expressed throughout all stages of gametogenesis in the embryo sac leaves of course the possibility open that they directly play a function in the female gametophyte. The employment of an embryo sac specific promoter could be useful in order to validate this hypothesis and to be able to better distinguish between the sporophytic and gametophytic roles of *REM34, REM35*, and *REM36*.

To understand how the *REM* genes under study act, we tested whether the down-regulations of *REM34*, *REM35*, and *REM36* perturbed the expression of genes known to be involved in gametogenesis progression. These genes were classified accordingly to their biological function in three categories: cell cycle control, chromatin remodeling, and ribosome biogenesis. Interestingly, we observed that several genes involved in different biological pathways were downregulated in the *REM_RNAi* lines. This observation suggests that *REM34*, *REM35*, and, in some measures, *REM36* are involved in the control of a very early steps of gametogenesis. In particular, they regulate the expression of different targets both directly and indirectly along the genetic network that controls gametophytic development in *Arabidopsis*.

Among the downregulated genes, the one that stands out most is *HAM2*, a HAT that, together with its homolog *HAM1*, belongs to the MYST clade of the HAT family and was shown to be involved in post-meiotic control of female and male gametophytic development (Latrasse et al., 2008). In mammals, the MYST protein family was found to be involved in many fundamental cell functions such as cell cycle progression and DNA repair (Pillus, 2008; Sapountzi and Côté, 2011). Furthermore, the human MYST4 acetylase was found to be expressed and involved in the control of gametogenesis as well (McGraw et al., 2007). In *Arabidopsis*, the *ham1 ham2* double mutant is lethal, while keeping one of the two genes heterozygous for the mutant allele resulted in a post-meiotic arrest of both female and male gametophyte developments (Latrasse et al., 2008). This phenotype is similar to the one observed in the *REM_RNAi* as well as in the *35S:REM34_EAR* lines. Interestingly, *HAM1* and *HAM2* were also found to be involved in the control of flowering time *via* the epigenetic regulation of *FLOWERING LOCUS C* (*FLC*), which is also a target of VRN1, one of the four *REM* genes for which the function is known so far, suggesting a common mechanism throughout plant development. The artificial silencing of these two acetyltransferases causes an early flowering phenotype (Xiao et al., 2013) which was also noticed in the *REM_RNAi* lines (data not shown). The downregulation of *HAM2* and the phenotypical similarity between the *REM_RNAi* lines and the *HAM* downregulation suggest that these genes might be involved in the control of the same biological processes throughout *Arabidopsis* development. Further analysis will be needed to confirm the possible interaction between *REM34*, *REM35*, and the HATs HAM1 and HAM2. The observed downregulation of the other analyzed target genes could be due to the general deregulation of transcription caused by the reduced expression of the chromatin remodeling factor *HAM2*.

While not much is known about the *REM* gene family, substantial information is available for other transcription factor families that are characterized by the presence of the B3 DNA binding domain. In particular, the well-characterized *auxin response factor* (*ARF*) family, known to play a crucial role in regulating auxin responses, and the *related to ABI3/VP1* (*RAV*) family, which was found to be involved in hormonal regulation during different stages of *Arabidopsis* development (Swaminathan et al., 2008). The plant hormone auxin was found to be involved in gametogenesis (Pagnussat et al., 2009; Panoli et al., 2015). Indeed, perturbation of auxin transport in the embryo sac causes an arrest in the earliest stages of megagametogenesis (Ceccato et al., 2013). Auxin biosynthesis in the male gametophyte was also recently shown to be essential for the transition from microsporogenesis to microgametogenesis (Yao et al., 2018). Because of the phenotypic similarities between the auxin defective mutant (Pagnussat et al., 2009; Panoli et al., 2015; Ceccato et al., 2013) and the *REM_RNAi* lines and because of the linkage between transcription factors containing the B3 DNA-binding domain and the regulation of hormonal responses, it is tempting to speculate that the role of *REM34, REM35*, and *REM36* play in gametogenesis is also based on the regulation of a hormonal related processes.

In summary, we gained new information about the expression pattern and function of *REM34, REM35*, and *REM36* during gametophyte development in *Arabidopsis*; those genes might control post-meiotic divisions in both embryo sac and pollen grains. These findings underline further the importance of *REM* genes during reproductive development in plants. Although these genes are often highly redundant and physically linked in the genome, slowly on, we start to get a better understanding about their functions in plant development. Of course, we just see the tip of the iceberg and still a huge amount of work has to be done to fully understand in detail the molecular and genetic mechanisms by which *REM* genes function.

## Data Availability Statement

All datasets generated for this study are included in the manuscript/**Supplementary Files**.

## Author Contributions

FC performed most of the experiments and wrote the manuscript. VB performed morphological analyses and contributed to writing the manuscript. OM and GL designed and employed the RNA interference and EAR constructs. MM designed and performed the CLS experiment and performed part of the back-crosses. RP made BiFC experiments. HH-U and SF designed the Y2H screening and helped FC with the experiment. AG designed the in-vitro pollen germination experiment and the Aniline blue analyses. MK contributed to the design of the experiments and helped writing the manuscript. VG designed the research, helped with the experiments and wrote the manuscript.

## Funding

VG was supported by Ministero dell’Istruzione, dell’Università e della Ricerca MIUR, SIR2014 MADSMEC, Proposal number RBSI14BTZR. The post-doctoral fellowship of AG was supported by MIUR, SIR2014 MADSMEC, Proposal number RBSI14BTZR.

The PhD fellowship of FC and RP were supported by the Doctorate School in Molecular and Cellular Biology, Università degli Studi di Milano. FC was supported by PROCROP-H20MC_RISE15LCOLO_M. RP was supported by H2020-MSCA-RISE-2015 ExpoSEED Proposal Number: 691109.

Work in the SF laboratory was financed by the Mexican National Council of Science and Technology (CONACyT) grants CB-2012-177739 and FC-2015-2/1061, and SF acknowledges support of the Marcos Moshinsky Foundation and the European Union H2020-MSCA-RISE-2015 project ExpoSEED (grant no. 691109).

## Conflict of Interest

The authors declare that the research was conducted in the absence of any commercial or financial relationships that could be construed as a potential conflict of interest.
