# Supplementary material for: REM34 and REM35 Control Female and Male Gametophyte Development in Arabidopsis thaliana
Source: Front Plant Sci. 2019 Oct 24;10:1351. doi: 10.3389/fpls.2019.01351 (PMC6821719; doi:10.3389/fpls.2019.01351)
Supplement: Supplementary file 1 [file Table_1.docx]

Supplementary Material

**Supplementary Figure 1**

qRT-PCRs, performed on three different *REM_RNAi* *#T2.1* plants, to evaluate the specificity of the three fragments chosen for the RNA interference construct. Two genes were selected: *REM37*, which was the possible off target of one of the fragments, and *REM39*, that was chosen due to the fact that is the *REM* gene with the higher expression in the tissues of interest belonging to the same cluster as *REM34, REM35* and *REM36* (Mantegazza et al., 2014)*.* Downregulation of neither *REM37* nor *REM39* was measured. The expression levels of *REM34, RERM35* and *REM36* were also measured, to make sure that the silencing of the target genes was maintained in the T2 generation.

**Supplementary Figure 2**

***REM_RNAi T2* lines have a reduced seed set compared to the wild-type**

(A) and (C) Graphs showing the mean number of ovules/silique in the wild-type and *REM_RNAi #1T2.4a* to *REM_RNAi #1T2.4j* and *REM_RNAi #1T2.5a* to *REM_RNAi #1T2.5j* plants resistant to the herbicide selection, divided in seeds and not fertilized ovules. Compared to the wild-type situation, in which each silique contains on average 50 ovules, the *REM_RNAi* siliques have on average 36.9 to 46.5 ovules. On average between 32.25% and 58.61% of ovules, depending from the analyzed line, failed to be fertilized, while no aborted ovules were detected in the wild type situation. Bars indicate the Standard deviation.

(B) and (D) For each line an evaluation of T-DNA abundancy in each of the 9 plants analyzed, is presented. The RT-PCR analyses shows a various amount of T-DNA amplicon which is clearly unrelated to the ovule abortions and the overall seed set in both *REM_RNAi #1T2.4* and *REM_RNAi #1T2.5* lines. The primers used are on ACTIN7 used as normalizer and the herbicide resistance gene BAR used to estimate the copy of T-DNA.


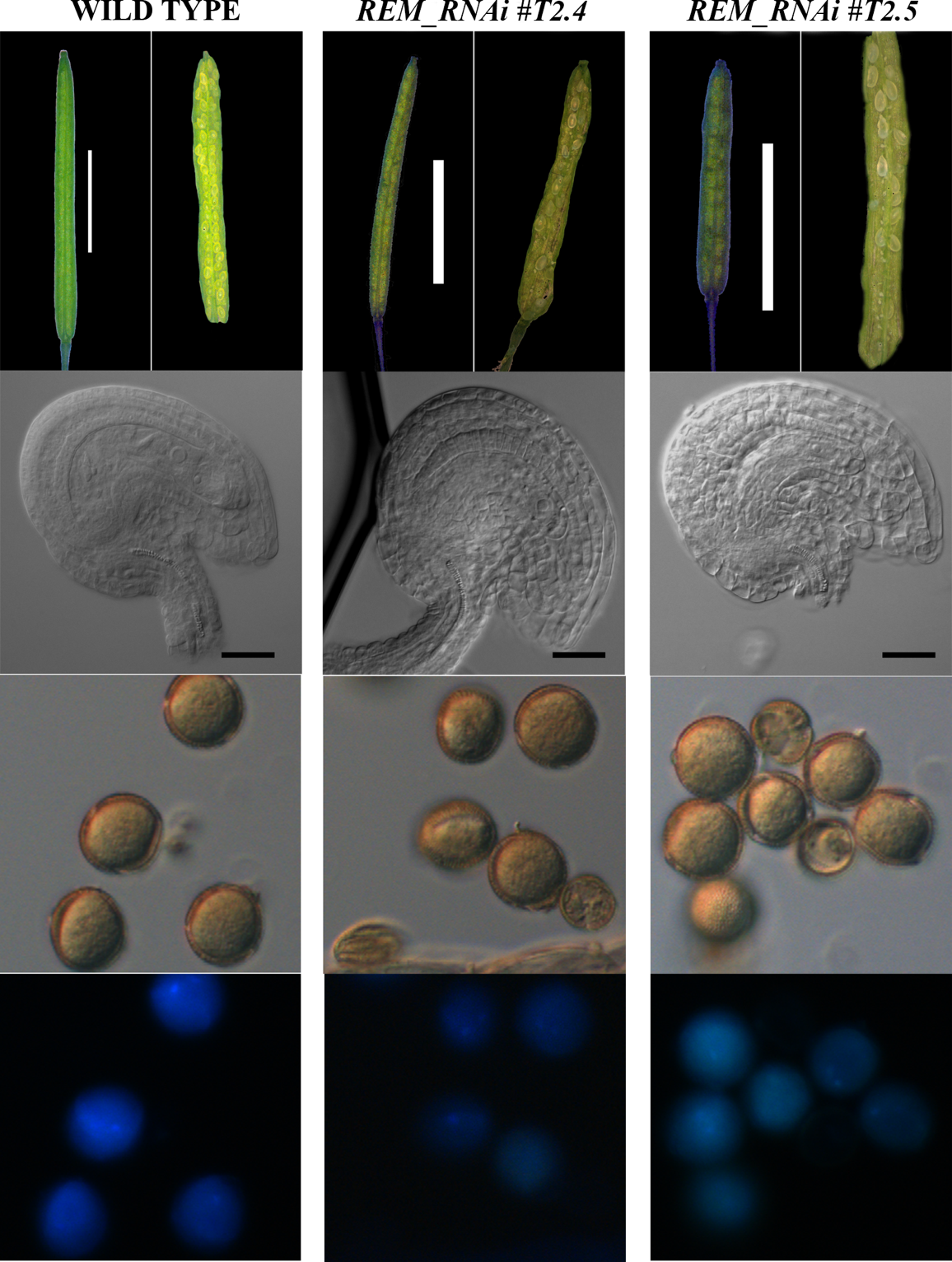


**Supplementary Figure 3**

Analysis of three different *REM_RNAi* T2 transgenic lines, all characterized by shorter siliques compared to the wild-type, a reduced seed set and ovule abortions. The majority of the embryo sacs of these lines showed a block in the earliest stages of megagametogenesis. Pollen collected from mature anthers is characterized by the presence of some degenerate grains, with no nucleus.

(White bar=5mm, black bar=20µm)


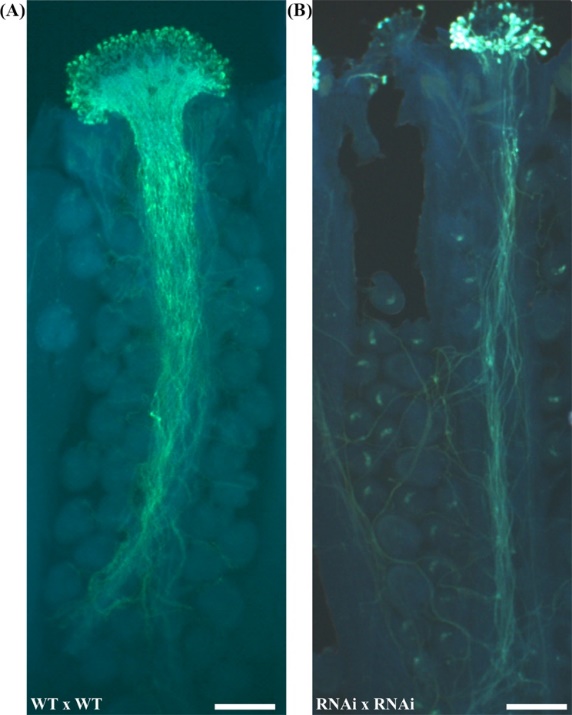


**Supplementary Figure 4**

*REM_RNAi #T3.*1 pollen germination test *in vitro,* showing a 30% decrease in the germination capability in the *REM_RNAi* line compared to the wild type.

Aniline staining was employed to visualize pollen grains adhesion to the stigma and pollen tube germination and growth. Compared to the wild-type situation (A), a lower number of REM_RNAi #1 grains were able to adhere to the stigma and to germinate (B). In both cases the pollen tubes grow correctly until the end of the transmitting tract, In *REM_RNAi #1T3.1*x *REM_RNAi #1T3.1* cross, several ovules are not targeted by a pollen tube correlated to the fact that about 40% of the ovules are blocked in FG1 stage and so synergids are not properly formed (Bar=100μm).


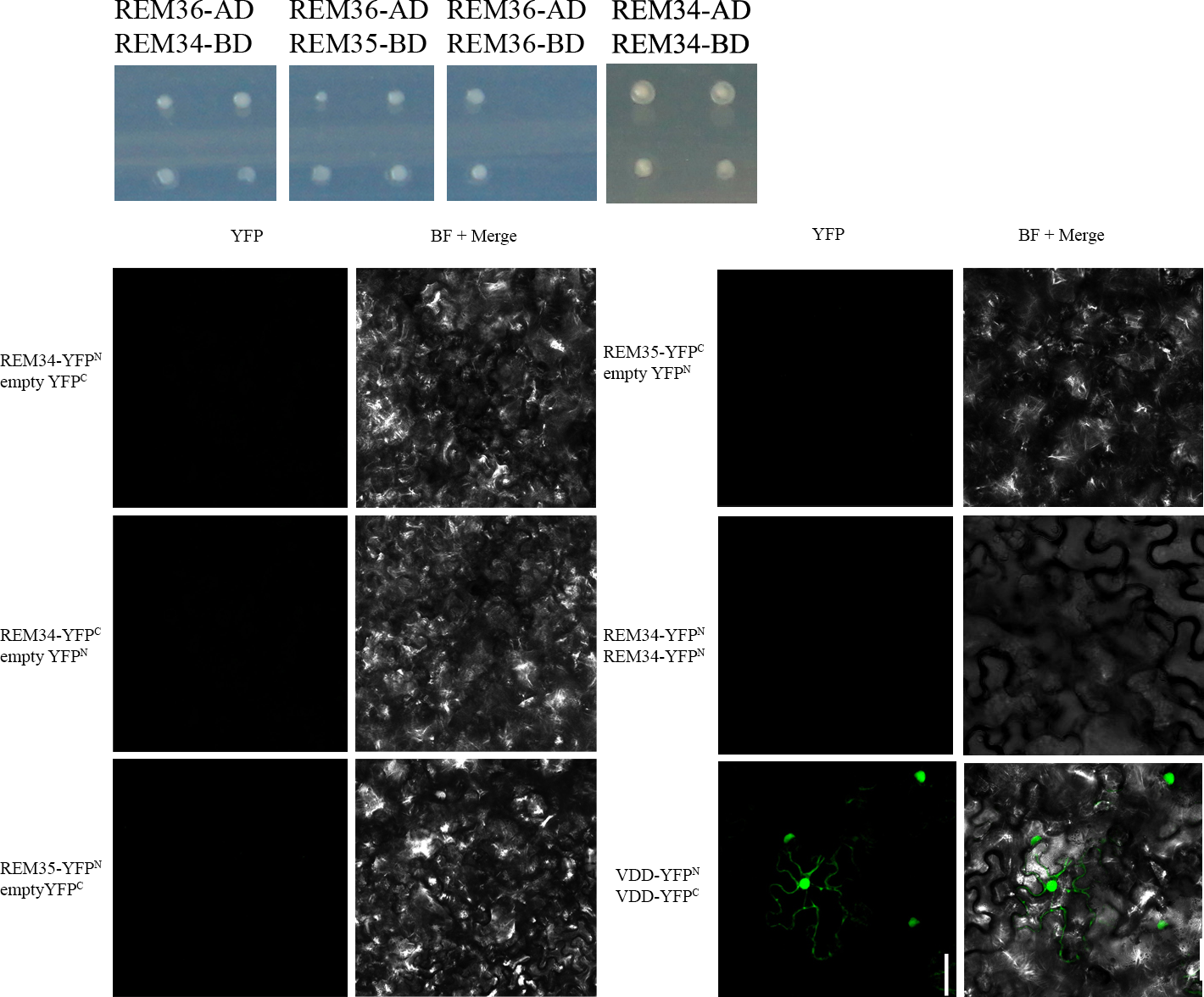


**Supplementary Figure 5**

In the Y2H assays, no interactions were detected for REM36 and REM34 homodimers.

Negative and positive controls for the BiFC experiment: all the constructs were cotranformed with the corresponding empty vector to test for false positive interaction. The REM34_REM34 interaction, which was found to be negative in the Y2H screening, was also employed as a negative control. Finally, as a positive control, the VDD_VDD interaction (M. Mendes et al, 2014) was tested.

**Supplementary Figure 6**

q-RTPCR on inflorescence on *REM_EAR #T2.1* and *#T2.7.* Compared to the wild type, none of the *REM* genes analyzed were found to be downregulated.

| **Supplementary Table 1: PRIMERS** |  |  |
| --- | --- | --- |
| GGTCTCACACCTGAAGTTTCCAAAGGAAAGG | REM34 FW golden gate cloning |  |
| GGTCTCTATCTCTCTCCAACCTCTTC | REM34 REV golden gate cloning |  |
| GGTCTCAAGATTCCAAGTCCAAGGACAAG | REM35 FW golden gate cloning |  |
| GGTCTCGTGTCAACAATAATCTGTTTC | REM35 REV golden gate cloning |  |
| GGTCTCAGACATCATCAAGTCTAGAAGGGAAG | REM36 FW golden gate cloning |  |
| GGTCTCGCCTTAATCATCCCACAAGCACAC | REM36 REV golden gate cloning |  |
| CACCATGGCGGATCCACCACATTTC | REM34 FW for REM34_EAR fusion |  |
| CTAAGCAAATCCAAGTCTAAGTTCAAGATCAAGATCAAGAACCAGATTACTGCTGAGG | REM34 RV for REM34_EAR fusion |  |
| **GGGGACAAGTTTGTACAAAAAAGCAGGCT**TCATGGCGGATCCACCACATTTCTC | REM34 FW for CDS cloning GW |  |
| **GGGGACCACTTTGTACAAGAAAGCTGGGT**TCAAACCAGATTACTGCTGAGG | REM34 REV for CDS cloning GW |  |
| **GGGGACAAGTTTGTACAAAAAAGCAGGCT**TCATGGATGATCCAGCAATTTC | REM35 FW for CDS cloning GW |  |
| **GGGGACAAGTTTGTACAAAAAAGCAGGCT**TCATGGCGAATCATCCACTA | REM36 FW for CDS cloning GW |  |
| **GGGGACCACTTTGTACAAGAAAGCTGGGT**CTTACTTGAGGATTTTGTTGATTTCCG | REM35/REM36 RV for CDS cloning GW |  |
| CACTCAGGTTTCATCACAGCACG | REM34 FW in situ probe | From Mantegazza et al., 2014 |
| **TAATACGACTCACTATAGGG**TAGCCCATTAGCGCAGCAGAAG | REM34 REV +T7 in situ probe | From Mantegazza et al., 2014 |
| TCCTATGTAGCTTCTGGCGATGG | REM35 FW in situ probe | From Mantegazza et al., 2014 |
| **TAATACGACTCACTATAGGG**AGTCTCCCCTCCTTCATCAAATGG | REM35 REV +T7 in situ probe | From Mantegazza et al., 2014 |
| CTCACTGCTTCCAACCTACG | REM36 FW in situ probe | From Mantegazza et al., 2014 |
| **TAATACGACTCACTATAGGG**AGCGTCCACGGATAAAAGCCTG | REM36 REV +T7 in situ probe | From Mantegazza et al., 2014 |
| AGCTTGTGAGACTGCTCCAC | REM34 FW expression analysis |  |
| CCTGATCGGAGACTGAGCAC | REM34 REV expression analysis |  |
| CATTTGATGAAGGAGGGGAGAC | REM35 FW expression analysis |  |
| CTTTCTAGCTCTGACCGAATCC | REM35 REV expression analysis |  |
| TCACTTGCTGGACACACCTC | REM36 FW expression analysis |  |
| TCGTCTCGAAGACAGTGTGC | REM36 REV expression analysis |  |
| TGGCATAGAGTGGAAGTCGCATC | REM37 FW expression analysis |  |
| GTCATTCGGGGTTTCCTATCC | REM37 REV expression analysis |  |
| GGAGAAGTTTCTGCCGTGAG | REM39 FW expression analysis |  |
| GGTCACTGGCCACTCTTCTC | REM39 REV expression analysis |  |
| GCAAGCTCAGTGGTGACTAC | MDS FW expression analysis |  |
| ACATCCACTTTCTGACATGC | MDS REV expression analysis |  |
| GTTAACCGTTGCTCACAGAC | NLE FW expression analysis |  |
| GCCTTTGCAAGTAAACAATG | NLE REV expression analysis |  |
| AGTCGCCTGCTGCTAAGACAAA | RBR FW expression analysis |  |
| ATGACAGTCCTGAGCCACTTGG | RBR REV expression analysis |  |
| CTTCTGATTCATCACCGGACTC | KRP6 FW expression analysis | From Liu J et al. 2008 |
| ACACCAAACGACGAACTGTTCT | KRP6 REV expression analysis | From Liu J et al. 2008 |
| ATGGGATCGTCTGCGGATACA | HAM1 FW expression analysis | From Latrasse et al., 2008 |
| GAATTCGTGAGAGCGAGTATCGCA | HAM1 REV expression analysis | From Latrasse et al., 2008 |
| CCTTTAACTCCTGATCAAGCTAT | HAM2 FW expression analysis | From Latrasse et al., 2008 |
| CTACAGCGCACTCTACTGAATC | HAM2 REV expression analysis | From Latrasse et al., 2008 |
| ATCTCGGTGACGGGCAGGACC | BAR gene FW |  |
| TCTACACCCACCTGCTGAAG | BAR gene REV |  |
| CGTTTCGCTTTCCTTAGTGTTAGCT | Actin 7 FW |  |
| AGCGAACGGATCTAGAGACTCACCTTG | Actin 7 REV |  |
